# Emergence of zoonotic *Brugia pahangi* parasite in Thailand

**DOI:** 10.14202/vetworld.2023.752-765

**Published:** 2023-04-14

**Authors:** Adisak Bhumiratana, Prapa Nunthawarasilp, Apiradee Intarapuk, Suntorn Pimnon, Wanapa Ritthison

**Affiliations:** 1 Thammasat University Research Unit in One Health and EcoHealth, Pathum Thani, Thailand; 2 Faculty of Public Health, Thammasat University, Pathum Thani 12121, Thailand; 3 Faculty of Public Health, Burapha University, Chonburi, Thailand; 4 Faculty of Veterinary Medicine, Mahanakorn University of Technology, Bangkok 10530, Thailand; 5 Faculty of Public Health, Bangkokthonburi University, Bangkok 10170, Thailand; 6 Office of Disease Prevention and Control, Region 6 Chonburi, Thailand

**Keywords:** *Brugia pahangi*, plantation-related zoonotic *Brugia pahangi* filariasis, sporadic transmission pattern, zoonosis

## Abstract

Zoonotic *Brugia pahangi* parasite infections in humans have emerged over two decades in Southeast Asia (SEA), including Malaysia and Thailand. The species is commonly found in domestic cats and dogs as the natural reservoir hosts. The sporadic transmission pattern of *B. pahangi* zoonosis causes childhood infections in Thailand and adulthood infections in Malaysia. It is crucial to understand the vulnerability in how zoonotic *B. pahangi* parasite is transmitted to susceptible persons in receptive settings and the exposure to the infection under impoverished environment to which the human-vector-animal interactions are related. This acquisition of knowledge will help multiple health science professions to apply One Health approach to strengthening the capacity in diagnosis and surveillance, and hence detecting and monitoring the “lingering” zoonotic *B. pahangi* infections present in vulnerable populations in Thailand and elsewhere in SEA. In this review article, the authors focused on articulating the concepts of plantation-related zoonotic *B. pahangi* filariasis by updating current knowledge of *B. pahangi* life cycle, vector’s life cycle and current state of research on the epidemiology and ecology of *B. pahangi* zoonosis.

## Introduction

*Brugia pahangi* is a mosquito-borne filarial nematode parasite, which was originally isolated from cats and dogs in Malaysia and identified as *Wuchereria pahangi* [1]. The species was then subsequently belonged to the genus *Brugia* by Buckley [2]. It is commonly found in domestic cats and dogs as the natural reservoir hosts [3–5]. In recent years, this parasite causes zoonotic infections in humans in Southeast Asia (SEA), including Malaysia [6, 7] and Thailand [8, 9]. The clinical presentations of human *B. pahangi* filariasis may vary with patient age due likely to host immune responses; that is, the kinetics of filarial infection and worm load may undergo the clinical, physiological, and immunological pathogenesis of lymphatic pathology as seen in cats [10] or dogs [11].

*Brugia pahangi* is genetically closely related to *Brugia malayi* [12]. Based on genomic analyses and gene annotation of *B. pahangi* draft genome (85.4 Mb) [13] that spans 9,687 protein-encoding genes, *B. pahangi* has high genomic similarity to that of zoonotic *B. malayi* parasite. *Brugia pahangi* possesses 8,681 predicted genes (89.6%) orthologous to *B. malayi*. Of note, 1624 genes are predicted to share exclusively similarity to *B. malayi* and 569 genes are unique to *B. pahangi*. The closely related genetic entity of *B. pahangi* and *B. malayi* implies that they share the biology of parasitic system of the interactions between these parasites and their vertebrate and/or invertebrate hosts. Nonetheless, it is evident that the physiology and vector competence of *B. pahangi* differ from *B. malayi* [8, 14–16].

In this article, the authors provide a review of the literature on *B. pahangi* and articulate ideas or concepts of plantation-related zoonotic *B. pahangi* filariasis by updating current knowledge of *B. pahangi* life cycle, vector’s life cycle, and current state of research on the epidemiology and ecology of *B. pahangi* zoonosis. Understanding the vulnerability of local people, that is, pertaining to plausible causes and consequences of human activities, relies on exploring the susceptibility of vulnerable persons in receptive settings and the exposure to the infection under impoverished environment to which the human-vector-animal interactions are related. This One Health approach would help multiple health science professions to understand veterinary public health, zoonotic disease, and the environment. More significantly, strengthening the capacity in diagnosis and surveillance is essential for detecting and monitoring the “lingering” zoonotic *B. pahangi* infections present in vulnerable populations in Thailand and elsewhere in SEA.

## Life Cycle of Zoonotic *B. pahangi* Parasite

**Figure-1:**
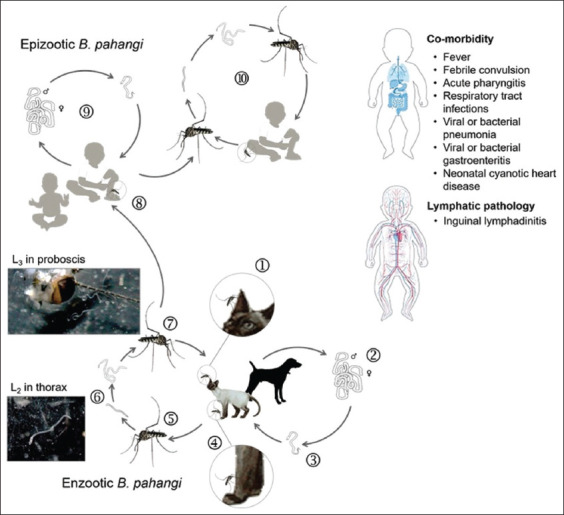
Life cycle of zoonotic *Brugia pahangi*. The parasite develops enzootic cycle of *B. pahangi* by which domestic cat or dog acquires the infection through mosquito-borne transmission ①-⑦. Transmission occurs when female adult of *Armigeres subalbatus* as a principal vector can transmit L_3_ infective stage ⑦ to susceptible cat or dog during taking blood meal ①. An infected cat or dog frequently exposed to infective bites can develop the adult worm infection ② and later microfilaremia ③. The complete cycle of transmission occurs when microfilariae are transmitted to susceptible female adult of *Ar. subalbatus* ⑤ that takes another blood meal from infected cat or dog ④. Microfilariae can develop further juvenile stages by exsheathment in midgut ⑤, and later L_1_ in hemolymph to L_2_ in thorax ⑥ to L_3_ in proboscis as infective stage ⑦. The parasite can also develop epizootic cycle of transmission ⑨ by which susceptible children acquires the accidental infection through *Ar. subalbatus* borne transmission ⑧. The childhood infection can undergo this epizootic *B. pahangi* by a single-step spillover ⑧. Meanwhile a spillback or human to human transmission of epizootic *B. pahangi* ⑩ remains unclear. [Source: Graphic illustration created by A. Bhumiratana].

The life cycle of zoonotic *B. pahangi* parasite illustrated in Figure-1 involves two transmission cycles, that are, enzootic and epizootic cycles. In enzootic cycle, enzootic *B. pahangi* parasite circulates among domestic dogs and/or cats as natural reservoir hosts through vertical transmission (between animal and vector hosts). In epizootic cycle, epizootic *B. pahangi* parasite occurs in humans as an accidental host through vertical transmission (between human and vector hosts). In given foci, the numbers of enzootic *B. pahangi* infections present in animal reservoirs (domestic dogs and/or cats) and local vector populations infer the endemicity as the degree to which transmission of epizootic *B. pahangi* occurs in susceptible person(s) in an impoverished environment of urban, sub-urban, or rural areas [3–7, 17, 18].

Transmission of enzootic *B. pahangi* parasites occurs when a susceptible dog or cat becomes frequently exposed to bites of local vectors, including infective bites. A diverse group of local vectors (*Mansonia*, *Aedes*, *Culex*, *Anopheles*, and *Armigeres subalbatus*) can play a possible role in transmission [8, 16, 19, 20] if they are adapted to local environment to which the animal reservoir–vector interactions are related. The infection occurs when an exposed dog or cat is inoculated with infective third-stage larvae (L_3_), which are released from the proboscis (mouth parts) of an infected mosquito while taking blood meal. The kinetics of the infection with L_3_ inoculum undergoes the localization and molting from L_3_ to fourth stage (L_4_) to adult. Incubation period takes about 23 days, for which the infection carries male worms and about 27 days for female worms [21]. Adult worm infection then produces microfilariae present in blood about 40–60 days or up to 3 months after the infection [22–24]. Individually infected dogs or cats with microfilaremic infection can harbor a broad range of microfilarial densities [25–27], showing microfilarial periodicity (i.e., the appearance of microfilariae with a peak density in blood in infected dog or cat during daytime or nighttime). Microfilarial periodicity is clinically unimportant but likely important for the epidemiologic implication that it relates its peak density to feeding behaviors of local potent vectors. Like *B. malayi* causing human lymphatic filariasis [28], adult worms of *B. pahangi* can live in cats or dogs for 2–5 years, and can cause a spectrum of clinical manifestations. Repeated infections (or with multiple inoculations of L_3_) are common during pre-patent and patent periods [29] and can influence the up-regulation of host immune responses and disease, for example, the clinical, physiological, and immunological pathogenesis of the structure and function of lymphatics in infected cats [10, 24, 30–32]. Therefore, microfilarial densities can vary with the age of infected cats or dogs due likely to prolonged exposure to the enzoonotic *B. pahangi* infections and individual immune responses to the infections [23, 28, 30]. To complete life cycle, *B. pahangi* microfilarial parasites circulating in the peripheral blood of an infected cat or dog can be transmitted to local potent vectors while taking blood meals. Similar to that of *B. malayi*, the extrinsic period of *B. pahangi* can take about 7–11 days [19], during which molting of microfilariae undergoes in body parts of infected mosquito, followed by migration of infective larvae into the proboscis after the ingestion of blood containing microfilariae [8, 33]. Infected mosquitoes can then transmit L_3_ to other dogs or cats when taking another blood meal.

*Brugia pahangi* is not only transmitted among the animal reservoirs (domestic dogs or cats) but also switching the animal reservoir hosts so-called “zoonotic spillover” [34, 35]. Zoonotic spillover requires intertwined interactions that can sustain transmission of enzootic parasites between the animal reservoir population (domestic dogs and/or cats) and the local vector population. The spillover transmission of epizootic *B. pahangi* parasites is generated by various factors and successive processes of multiple spillovers that enable them to establish the infection in a susceptible person through frequent exposure to multiple bites of local potent vector(s) carrying the infection in home or work locations. Thus, if accompanied by poor living conditions and the lack of preventive measure or behavior, a susceptible person, especially an infant and preschool-age child, is more likely to become frequently exposed to the infection in indoor setting in home location than an outdoor setting. As compared to other indoor-resting mosquitoes like *Aedes aegypti* and *Culex quinquefasciatus*, *Ar. subalbatus* is a peri-domestic species that rests outdoors but more likely it has the potential to transmit epizootic *B. pahangi* parasites in impoverished environment of semiurban and rural areas in Malaysia [6, 7, 19] and Thailand [8, 9, 17, 18]. Adulthood infections are evident in Malaysia [6, 7, 19]. The “lingering” childhood infections (under 2 years of age) rather than adulthood infections are sporadic in Thailand [8, 9]. Such preschool-age children who carry the epizootic *B. pahangi* infections may harbor a wide range of microfilarial densities in the presence or absence of lymphatic pathology [8]. More likely, they develop the co-morbidity (Figure-1) in their lifetime in pre-patent or patent period. As is compared to *B. pahangi* or *B. malayi* infected cat, the incubation period for childhood infections is estimated as early as 3 months or up to 6 months [8]. The incubation period for adulthood infections remains unclear. The extrinsic period of zoonotic *B. pahangi* parasite in *Ar. subalbatus* is estimated 7–11 days [19]. Susceptible persons acquire repeated infections with zoonotic *B. pahangi* parasites (or multiple spillovers of epizootic strains) through mosquito-borne transmission – nor are they protected by any preventive measures or behaviors. The establishment of *B. pahangi* infection in *Ar. subalbatus* is later mentioned in detail.

## Diagnosis of Zoonotic *B. pahangi* Parasite

Parasitological approaches to diagnosing infections in animal reservoirs and vectors are based on several methods of the preparation, detection, and identification of diagnostic stages of *B. pahangi*, that is, detecting and identifying morphologic characteristics of microfilariae and adult worms in cats or dogs and L_3_ in mosquitoes. The adult worms are found in the lymphatic vessels and nodes in infected cat or dog. Microfilariae are found in blood vessels in infected cat or dog. L_3_ larvae are found in proboscis of infected mosquito. It is clear that, in experimentally animal models such as cats [36] or ferrets [37, 38], L_3_ larvae of both *B. pahangi* and *B. malayi* can mature into adult worms in the popliteal and inguinal lymphatics of the injected limb with L_3_ inoculations and later microfilariae released by the fertilized female adult worms can live in blood circulation from 3 to 8 months after infection. Jackson-Thompson *et al*. [38] demonstrated that in ferrets, about 90% of adult worms were found in the inguinal and femoral lymphatics draining the infected limb and relatively less amount in the draining lymphatic vessels of the contralateral side. After 3–8 months’ post-infection, all infected ferrets developed microfilaremia with a peak density between 16 and 20-weeks post-infection. However, parasitic worm burdens, that is, both adult worms and microfilariae carried by infected male and female ferrets, did not differ from each other.

The adult worms of *B. pahangi* are morphologically recognized, with only the males, but not females, and are distinguishable from other *Brugia* species. The male worms of *B. pahangi* are 13–17 to 20–23 μm long; the female worms are 38–43 to 55–63 μm long [2, 21]. The male worms of *B. pahangi* recovered from infected cats and dogs are very similar to *B. malayi* [2, 39] and *Brugia patei* [40, 41]; the difference is that they have shortest spicules. The left spicule is 200–215 μm long. The right spicule is 75–90 μm long. The male spicules of *B. patei* are intermediate. The left spicule of *B. patei* male worm is 270 μm long, and the right spicule is 116 μm long. The longest male spicules of *B. malayi* are also easily recognized. The left spicule of *B. malayi* adult male is 390 μm, whereas the right spicule is 125 μm long.

The microfilaria of *B. pahangi* is not easily distinguishable from other *Brugia* species [28, 42], but it is morphologically recognized upon the methods of preparation, staining, and identification. As is similar to that of *B. malayi*, the Giemsa-stained microfilaria microscopically visualized differs only in having a slightly short cephalic space [37, 43] but likely having a relatively long innenkorper [37, 44]. The body length of *B. pahangi* microfilaria is 270–290 μm long when examined by Knott’s concentration technique; and 180–200 μm long when examined by Giemsa-stained thick blood films [37]. When stained using the acid phosphatase histochemical method [45–47], the *B. pahangi* microfilaria is relatively red throughout the length of its body. By contrast, the microfilaria of *B. malayi* is red, mainly at the excretory and anal pores.

As is compared to *B. malayi* [37, 48] and *Wuchereria bancrofti* [49], the L_3_ larvae of *B. pahangi* can be found in some susceptible mosquitoes, that is, either experimental mosquitoes that artificially fed on microfilariae infected blood or wild-caught mosquitoes in the fields. The susceptibility of some vectors for *B. pahangi* is described later. It is believed that after post infective stage development of L_2_ in thoracic muscle of the susceptible mosquitoes, the majority of mature L_3_ larvae migrate into the proboscis and live several days or up to 36 days, or as long as the mosquito host dies. The morphology of infective L_3_ of *B. pahangi* is not easily distinguished from that of *B. malayi*. The body length of L_3_ larva of *B. malayi* is 1600–3000 μm long [48]. The morphology of L_3_ of *B. malayi* is well recognized; the tapered head bearing eight submedian papillae arranged in two circles and a pair of lateral amphids; the long cylindrical buccal capsule; and the cuticular lappets on the tail extremity. The sex of L_3_ larva can be identified in late L_2_ stage to mature L_3_ stage upon the position of the genital primordium. The genital primordium is located at the mid-esophagus in mature L_3_ female larva. It is located at or just posterior to the esophago-intestinal junction in mature L_3_ male larva.

Routine laboratory diagnosis of *B. pahangi* infections in animal reservoirs or humans relies upon standard microscopic methods of detecting and identifying the microfilaremic infection. As is compared to that of other filarial worms such as *Dirofilaria* spp., *B. malayi*, and *W. bancrofti*, Giemsa-stained sheathed microfilariae of *B. pahangi* are microscopically recognized at 100× to 400× magnification. The preferred method is the collection of paired blood samples obtained from clinical cases (cats, dogs, or humans) at time intervals (day and night). In particular, the daytime microfilaremia of *B. pahangi* found in a case of human *B. pahangi* filariasis should be followed-up by examining the nighttime microfilaremia. If the sub-periodic strains of *B. pahangi* are assumed, the microfilariae may appear in peripheral blood during daytime or nighttime as this microfilarial periodicity may differ the appearance in blood in infected cats or dogs.

**Figure-2:**
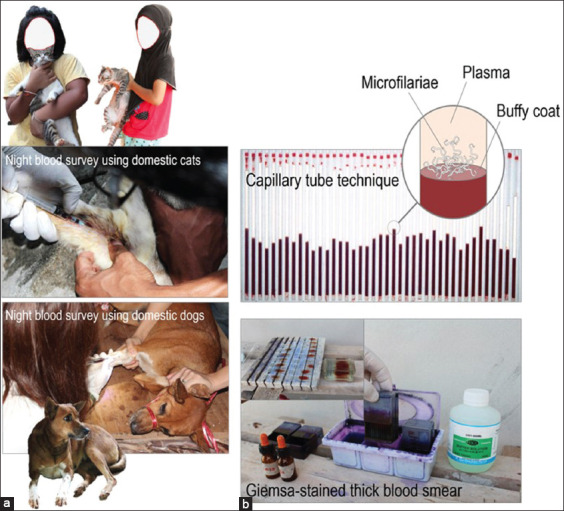
Animal reservoir survey using domestic cats and/or dogs. (a) During house-to-house visit, one collection of 1–2 mL blood volume of a domestic cat or dog is obtained by venipuncture at a time close to a peak hour of nocturnally sub-periodic microfilariae of *Brugia pahangi*. The cat or dog appropriately restrained by field staff is placed in either ventral or lateral recumbency with the forelimb (cephalic vein access) or hindlimb (saphenous vein access). The skin over the collection site that may or may not be clipped with an electric clipper is cleaned with 70% alcohol. (b) Standard microscopic blood examination using capillary tube technique or Giemsa’s stained thick blood smear can be applied under field conditions for screening or diagnosing *Brugia* spp. parasite infections. [Source: Graphic illustration created by A. Bhumiratana].

The endemic countries implementing the national program to eliminate lymphatic filariasis do not yet establish surveillance system for detecting and monitoring the zoonotic *B. pahangi* infections in animal reservoirs, nor do they ignore this neglected *B. pahangi*. Parasitological approaches applied to or used in case surveillance for human *B. pahangi* filariasis rely upon standard microscopic diagnosis of *B. pahangi* microfilaremic infections in cats or dogs of clinical filariasis (Figure-2). The preferred methods include thick blood films (using 20–60 mL blood of the ear vein, as well as cephalic, saphenous or jugular veins) [4, 5, 17, 18, 47, 50] and other specific and sensitive methods such as Knott’s concentration technique (using 1 mL blood) and membrane filtration (using 1–2 mL blood). However, the most preferred method is the application of polymerase chain reaction (PCR) assays [6–9, 17, 18, 46, 47, 50–52], which are highly specific and sensitive for *B. pahangi* distinguishable from other filarial parasites present in animal or human hosts or *Ar. subalbatus* vector [6-8] but not many other mosquitoes [53] as described below.

**Figure-3:**
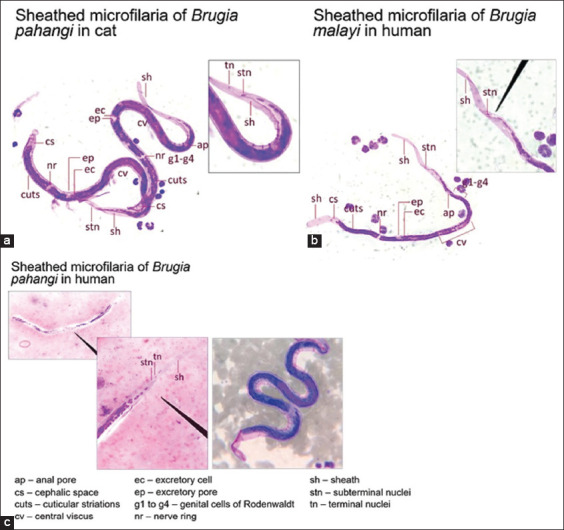
Comparison of well-defined morphologic characteristics (400× magnification) of nocturnally sub-periodic strains of *Brugia pahangi* and *Brugia malayi*. Giemsa-stained thick blood film as standard microscopic diagnosis of microfilarial parasites isolated from different sources of infection [8]: *B. pahangi* in cat (a) and human (c) and *B. malayi* in human (b). [Source: Graphic illustration created by A. Bhumiratana].

**Figure-4:**
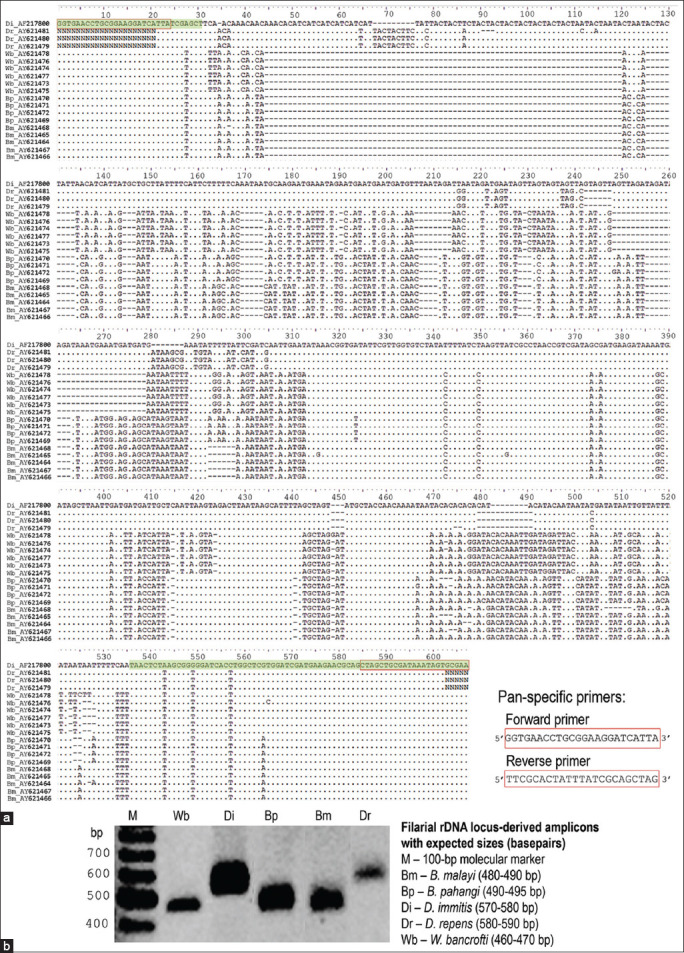
Polymerase chain reaction (PCR) using filarial ribosomal DNA internal transcribed spacer region 1 (ITS1) region-specific primers. (a) Multiple sequence alignment of amplified DNA fragments authentically derived from ribosomal genes of filarial parasites, which span partially 18S ribosomal RNA gene (green color-highlighted)-completely ITS1-partially 5.8S ribosomal RNA gene (green color-highlighted). The retrieved nucleotide sequences (accession no.) include *Brugia malayi* (Bm) (AY621464 to AY621468), *Brugia pahangi* (Bp) (AY621469 to AY621472), *Wuchereria bancrofti* (Wb) (AY621473 to AY621478), *Dirofilaria repens* (Dr) (AY621479 to AY621481), and *Dirofilaria immitis* (Di) (AF217800), whose DNA sequence data were derived from the GenBank database. BioEdit version 7.2 software application is used in multiple sequence alignment. The gap (insertion/deletion) is generated to maximize the homology representing conserved (•) and degenerate nucleotide residues. (b) PCR amplification using filarial ribosomal DNA ITS1 region-specific primers can yield putatively amplicons with expected sizes in reactions containing microfilarial DNA templates isolated from different sources of infections. [Source: Graphic illustration created by A. Bhumiratana].

Contrary to Malaysia, which reported the zoonotic *B. pahangi* micorfilaremic infections in clinical adult patients between 2003 and 2004 [6, 7], Thailand has reported cumulatively clinical microfilaremic cases in recent years; four patients under 2 years of age in Eastern and Southern Thailand [8] and a 64-year-old female adult patients in Central Thailand [9]. All clinical cases are considered sporadic infections with the microfilaremic state occurring outside transmission areas of *B. malayi*. The investigation of these clinical cases of zoonotic *B. pahangi* infections contemporarily occurred in SEA relies conventionally on standard microscopic methods, and subsequently, the infections are confirmed by molecular detection methods. For instance, Giemsa-stained thick blood films examined for the presence of *B. pahangi* microfilariae in the patient and cat are illustrated in Figure-3. The sheathed microfilariae of zoonotic *B. pahangi* parasites found in two cumulative children-patients in Rayong, Eastern Thailand (Figure-3c) [8] compared well to that commonly found in microfilaremic cat (Figure-3a) and to that isolated from a *B. malayi* infected patient living in *B. malayi* endemic area of Southern Thailand (Figure-3b). In addition, laboratory-confirmed investigation of zoonotic *B. pahangi* infections in children-patients [8] or adult patients [6, 7] relies on molecular marker-based PCR assays specific for detection of *Brugia* spp. parasite infection and/or *B. pahangi*. There are several candidate filarial orthologous genes as molecular markers, for example, *β-tubulin* [8], the mitochondrial 12S ribosomal RNA (i.e., the mitochondrial small and large ribosomal subunits contain 12S and 16S rRNAs encoded by mitochondrial DNA) [9], and *COX* I (i.e., mitochondrial cytochrome c oxidase 1-encoding gene) [7, 19]. In Figure-4, PCR amplification of the internal transcribed spacer region 1 (ITS) I, which is the highly variable region of rRNA genes, can provide proof that the *B. pahangi* infection is discriminated from other filarial parasite infections present in any sources of the infection. But PCR amplification pattern of this genetic marker does not explain the zoonotic spillover of *B. pahangi* parasite infections present between infected persons and animal reservoirs. Analysis of sequenced amplicons authentically derived from ITS I is required for further exploring the human carrying *B. pahangi* infection whether or not epidemiologically linked to animal reservoirs carrying *B. pahangi* infection in any foci.

In addition, antibody capture assays can be applied to the detection of anti-filarial immunoglobulin G4 (IgG4) antibodies present in serum or plasma samples of children patients or adult patients who are infected with zoonotic *B. pahangi* or other *Brugia* spp. parasites. Based on detection of specific IgG4 antibodies against BmR1 (*Brugia* Rapid) and BmSXP recombinant antigens [54–59], the commercially available antibody tests for brugian filariasis infection include the Brugia Rapid™ test (Reszon Diagnostics International Sdn. Bhd., Selangor, Malaysia) (https://reszonics.com/products/infectious-diseases-diagnosis/lateral-flow-rapid-test/*Brugia*-rapid-test/), and the PanLF Rapid™ test (Reszon Diagnostics International Sdn. Bhd.) (https://reszonics.com/products/infectious-diseases-diagnosis/lateral-flow-rapid-test/panlf-rapid-test/).

## Vector of Zoonotic *B. pahangi*

*Armigeres subalbatus* (Coquillett, 1898) (*Diptera*: *Culicidae*) belonging to the genus *Armigeres* is originally a forest-associated zoophilic mosquito [8, 19, 20]. It is geographically distributed throughout South Asia, East Asia, and SEA. Two forms of *Ar. subalbatus* that share physiology and common characteristics are forest and plantation ecotypes. Forest ecotype is adapted to local habitats in forests at different altitudes, such as coastal forests [60], tropical forests, and deciduous forests, but unlikely swamp forests [8] and mangrove forests [53]. Plantation ecotype can breed in plantation areas [9, 53, 61], including rubber trees, oil palms, and fruit orchards, as well as rice fields [62, 63]. The species is closely associated with human settlements with poor sanitation [8, 53, 61, 62], and can thrive in rural and sub-urban areas [64–67]. As compared to other *Aedes togoi* and *Cx. quinquefasciatu*s [16], it is a relatively potent vector of *B. pahangi* and *Dirofilaria immitis* [8, 17, 18], as well as other viruses [64–66], in human settlement areas. Perhaps it has the potential to transmit these zoonotic parasites temporally and spatially in receptive environments [8, 17, 19, 20]. Recently, there has been a line of evidence that the occurrence of human *B. pahangi* filariasis in Malaysia and Thailand has been epidemiologically linked with vector competence of *Ar. subalbatus* [7–9, 19]. Understanding vector competence of zoonotic *B. pahangi* parasite relies upon understanding its life cycle and success in adaptation of the plantation ecotype of *Ar. subalbatus* to human settlements.

**Figure-5:**
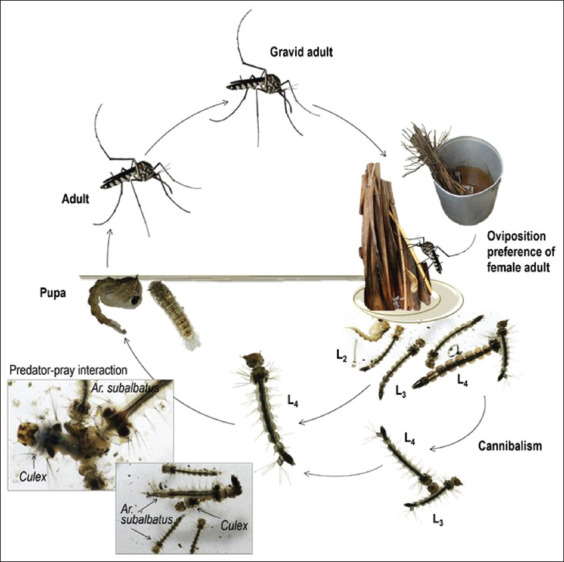
Life cycle of *Armegires subalbatus* for both forest and plantation ecotypes. [Source: Graphic illustration created by A. Bhumiratana].

**Figure-6:**
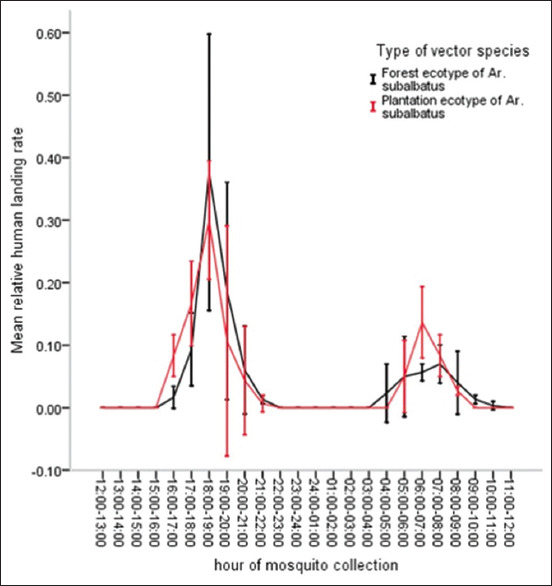
A 24 h cycle of biting activity of *Armegires subalbatus*. Biting activity of a given population of *Ar. subalbatus* represents mean relative human landing rate (HLR) (± 1 standard error denoted as bar) for each hour of mosquito collection by using 3 geographically defined population samples of each ecotype that were obtained from 3 different plantation areas connecting to forest fringes in Suratthani, Trat, and Rayong provinces. Relative HLR infers the number of human blood-seeking *Ar. subalbatus* female adult mosquitoes per night per person at each hour of mosquito collection (or HLR), which is divided by a sum of HLR for 24-h mosquito collection for each ecotype. Both forest and plantation ecotypes of *Ar. subalbatus* tend to seek human blood meals with a major peak hour (1800–1900 h) and another minor peak hour (0600–0700 h). [Source: Unpublished data by the authors].

Figure-5 illustrates a complete life cycle of the plantation ecotype of *Ar. subalbatus* that is constituted of adult and larval phases. It has four distinct developmental stages during its complete metamorphosis (i.e., egg, larva, pupa, and adult). Similar to other *Mansonia*, *Aedes*, *Culex*, and *Anopheles* [20, 68], female adult mosquito of *Ar. subalbatus* has a life span 1 month or up to 1.5 month during which its gonotrophic cycle (i.e., a fecundic life span) typically requires a blood meal by feeding on vertebrates (animals or humans) [60, 68, 69]. To obtain excess amount of blood as a source of a dietary supplement that stimulates and supports egg development, it is normally engorged. The number of its gonotophic cycle, as well as the number of laid eggs per gonotrophic cycle, is unknown. The duration of the gonotrophic cycle of *Ar. subalbatus* female adult mosquito delineates biting cycle (i.e., the frequency of vector–host contact) and potential transmission (i.e., the estimated number of infective L_3_ inoculation for transmission) of zoonotic *B. pahangi* parasite [8] during its life span. Two distinct circadian rhythms of female adult mosquitoes can be regulated by two different phases of the moon. Then biting activities occur when host-seeking behaviors increase during which the moon waxes as they decrease during which the moon wanes. As compared to other night-biting mosquitoes [68, 69], *Ar. subalbatus* elicits two biting cycles; highest peak of biting activities occurs early after the sunset or at dusk (18:00–20:00 h) [8, 68, 69] and a lesser peak at dawn (05:00–07:00 h) (Figure-6). Unlike the plantation ecotype, the forest ecotype of female adult mosquitoes actively seeks animal blood meals throughout the day in the forest.

**Figure-7:**
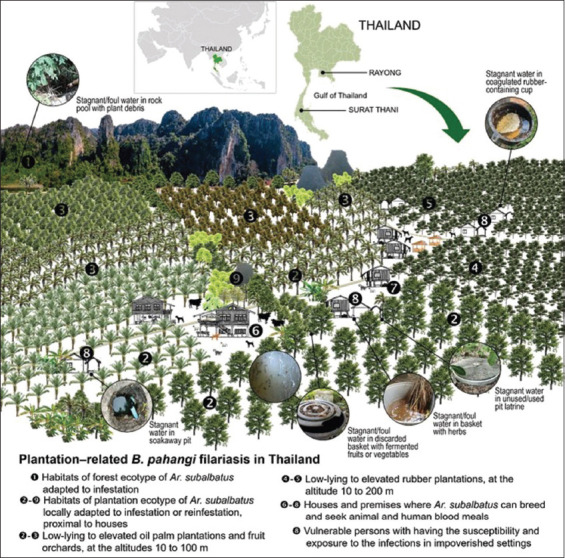
Pathogenic landscape of rural ecosystem of plantation-related *Brugia pahangi* filariasis in Thailand. This pathogenic landscape delineates complex eco-epidemiological settings in which vulnerable persons become close contact with animal reservoirs and *Armegires subalbatus* vectors under impoverished environments. [Source: Graphic illustration created by A. Bhumiratana].

The gravid female adult mosquito oviposits in naturally or artificially breeding sites containing stagnant foul waters (strongly polluted) with high organic contents [8, 70, 71]. The oviposition occurs during the nighttime. Most preferred natural containers include rock pools, tree holes, bamboo stumps, and banana stumps. Most preferred artificial containers include septic tanks, soakaway pits (drainages from households, rubber processing, fermented rice water, or fermented food waste), flower or plant holding baskets, fruits fermented tanks, and coagulated rubber containing cups (Figure-7). Contrary to oviposition, egg hatching, and adult eclosion occurs exclusively during the daytime. Larvae may be carnivorous [72] if particulate predator-prey interaction or cannibalism exists in food resource limited habitats. Larval molts (L_1_ to L_4_ stages) and pupation occur at night. Larval development from L_1_ to pupa takes about 10–14 days in suitable habitats.

## Plantation-related *B. pahangi* Filariasis

Plantation-related *B. pahangi* filariasis (Figure-7) is the illness attributed to the infection of zoonotic *B. pahangi* parasites [8] that occurs in a susceptible person through mosquito-borne transmission in a receptive setting of the plantations mixed with human settlements. A focus of infection is a place where establishing impoverished human settlements and sustaining the infections of zoonotic *B. pahangi* parasites present in the animal reservoir hosts (domestic cats and/or dogs) and the local vectors. Both animal reservoir and vector hosts are the source of the zoonotic *B. pahangi* infections which in turn generates multiple spillovers of epizootic *B. pahangi* parasites over space and time. In Figure-7, a comprehensive picture of plantation-related *B. pahangi* filariasis can be depicted with the view of a scenic pathogenic or disease landscape [73], which integrates landscape attributes of rural ecosystem, that is, the spatial extent of, or interactions between, plantations, human settlements, animal reservoir hosts, local vectors, and local agri-environmental climatic conditions [74–76]. The study of this pathogenic landscape of zoonotic *B. pahangi* filariasis relies on using methods and tools used either in landscape epidemiology (the spatial and temporal variation in disease risk or incidence) [76, 77], landscape ecology (the relationships between ecological processes in the environment and particular disease ecosystems) [78–82], or the integration of landscape epidemiology and ecology.

In practical, disease landscape is a geographically defined landscape of a unique rural ecosystem of plantation-related *B. pahangi* filariasis, especially as the result of unique effects of spatial heterogeneity on the intertwined human-vector-animal reservoir interactions as mentioned earlier. It can be defined as small as georeferenced land unit that is the basis of the land use map legend which integrates the topological features (altitude, slope, and curvature) and the various land attribute data (land-form, soil, water body, and vegetation) as is shaped by natural and/or human alterations of land use and land cover [81–84]. The driving forces and processes of human-induced land use change are more likely to be influenced by land management strategies than natural environmental change. Disease landscape in rural ecosystem which, in turn, regulates such emerging *B. pahangi* infections is shaped by human alterations of land use and land cover as the result of ecological processes of such driving forces such as human settlements, crop plantations, and domestication of livestock and pet animals [77, 82, 83].

Such landscape ecology and epidemiology of zoonotic *B. pahangi* are crucial to better understand the vulnerability in how multiple spillovers of zoonotic *B. pahangi* parasites occur in a rural setting with environmental determinants in Thailand as shown in Figure-7. The environmental determinants of plantation-related *B. pahangi* filariasis are, therefore, all external factors and conditions that affect people’s lives, especially impoverished people and children. In rural setting, vulnerable young children experience insect or mosquito bites frequently, especially when they are exposed while sleeping inside or outside houses in the absence of preventive measures. Furthermore, many people keep dogs to guard their homes or farms; and likely keep cats to catch rats. These family-pet animals are kept or taken care of for companionship or for personal safety, but the pet owners are not aware of animal reservoirs for zoonotic *B. pahangi* parasites that potentially spread the parasites to people.

Current evidence supports the fact that particularly in Malaysia [6, 7, 19] and Thailand [4, 5, 8, 9, 17, 18], local people did not know how communicable zoonotic *B. pahangi* parasite is, nor were they aware of the parasite being one of the most common zoonotic diseases of cats or dogs. Pet cats or dogs can pose a minimal zoonotic risk to their human companions. Although monitoring any sign of illness or disease in sick cats or dogs, the disease is not normally diagnosed and treated promptly due to the limitations of the owners’ caring. However, a person with a compromised immune system from disease or medication may render him or her slightly vulnerable to contracting zoonosis from his or her infected cat or dog. If accompanied by getting sick and of more serious illnesses or co-morbidities (Figure-1), a vulnerable person such as infants and preschool-age child has a higher chance of contracting the zoonotic *B. pahangi* infection [8] due likely to their immune compromise and the lack of family perception and awareness of such zoonotic disease and local vectors. Thereafter, such pathogenic landscape of plantation-related *B. pahangi* filariasis seen in Thailand strongly relates childhood infection or disease to the multiple spillovers of epizootic *B. pahangi* parasites in an infection pocket to which children-animal-vector interactions are related.

The infection pocket is explained by the establishment of “lingering” infection with, but not the propagated outbreak of, epizootic *B. pahangi* parasites. Such vulnerable person contracting zoonosis is likely to be epidemiologically linked with environmental determinants, for example, being frequently exposed to multiple bites of *Ar. subalbatus* in indoor setting in the absence of preventive measures. If accompanied by keeping close contact with infected cats or dogs and *Ar. subalbabtus* vectors over a period of time, such vulnerable person becomes at higher risk of, or is affected with, the infection of zoonotic *B. pahangi* parasites. This may be a reason why transmission pattern of zoonotic *B. pahangi* filariasis seems not to be the propagated outbreak as seen in transmission areas of other vector-borne diseases such as *W. bancrofti* filariasis [85, 86] and malaria [87, 88]. The establishment of such infection pocket in a pathogenic landscape as shown in Figure-7 relies on sustaining the prevalence and distribution of the infections with zoonotic *B. pahangi* parasites present in both cats and/or dogs and *Ar. subalbatus* vectors [8]. Taken together, this acquisition of knowledge of plantation-related *B. pahangi* filariasis is crucial for surveillance and case investigation, as mentioned below.

## Perspective in Surveillance and Case Investigation of Epizootic *B. pahangi* Infection

The occurrence of sporadic infection with epizootic *B. pahangi* parasites can be differentiated from that observed by local transmission caused by *B. malayi* or *W. bancrofti* in endemic settings. However, the detection of anomalous infection with epizootic *B. pahangi* parasites among vulnerable persons residing in *Ar. subalbatus* infested land areas does not rely on routine background surveillance as part of lymphatic filariasis control or elimination. For instance, surveillance and case investigation of epizootic *B. pahangi* infection among children-patients in Thailand [8] is based on a systemic collection of data and/or information obtained by the following methods such as medical record review, household survey, animal reservoir infection survey, and entomological survey.

### Medical record review

The medical record can provide information from when the patient was living, undergoing clinical symptoms and receiving differential diagnosis and empirical treatment by practitioners to their recovery. A medical record review could help the epidemiologists, infection control personnel, and public health professionals understand what caused the overt features of lymphatic pathology in the presence or absence of the co-morbidities [8], as shown for children-patients in Figure-1, or certain medical conditions. Recently the emergence of zoonotic *B. pahangi* infections in Thailand has revealed that the parents’ perceptions of current health problems of their children-patients may regard illnesses that are not associated with lymphatic filarial infections. More obviously, such cumulative children-patients were hospitalized with clinical presentations, which are not associated with lymphatic filarial infections [8]. On the other hand, empirical treatment of children-patients’ illnesses relies radically on epidemiological data and differential diagnosis whether clinically, parasitologically, or serologically. All microfilaremic infections were confirmed using PCR assays as mentioned earlier.

Moreover, the medical record review can provide further information of which the practitioners were diagnosing lymphatic filarial infection and the details of the eligibility criteria for treatment of children-patients with lymphatic filarial infection and treatment outcomes, that is, whether patho-physiologically, psychological, physically, or socially, that reflect favorable or adverse effects on the patients’ health and well-being. Lymphatic filarial infection often occurs in early childhood, but manifestation of clinical lymphedema typically occurs later in life. However, the detection of lymphatic obstruction in asymptomatic children has been documented using lymphoscintigraphy. *Brugia pahangi* in childhood infections does not seem to present overt features of lymphatic pathology. Similar to *B. malayi*, the infection can be treated with a single oral-dose 6 mg/kg diethylcarbamazine as anti-filarial drug, and microfilaremia clearance is observed through a course of radical treatment that can last for several months.

### Household survey

A house-to-house visit can provide information about which home environments are unsafe, why parents or caregivers of children-patients are unaware of protecting local vectors’ bites, and which living conditions render children-patients frequently exposed to biting of local vectors or susceptible to contracting *B. pahangi* zoonosis. Both unsafe home environment and unclean household environmental cleaning observed between a patient’s house and nearby houses are among plausible factors that influence the vulnerability of zoonotic *B. pahangi* infection.

### Animal reservoir infection survey

Children-patients contracting *B. pahangi* zoonosis are likely to be associated with keeping close contact with domestication of pet animals such as cats or dogs. In fact, a patient’s house may or may not keep any pet animals, but there is the preferred domestication of cats or dogs in neighboring houses. As mentioned earlier, animal reservoir infection survey using day or night blood samples of cats and/or dogs (Figure-2) can provide accurate data on the source of infection in an infection pocket, or within a 10–100-m radius of a patient’s house. The *B. pahangi* infection prevalence (or microfilaremia rate) determined by the preferred blood examination methods (Figure-2) is considered a proxy measure of sustaining *B. pahangi* zoonosis in a responsible infection pocket.

### Entomological survey

An entomological survey can provide the proof that the local vector has the potential to transmit *B. pahangi* zoonosis or carries the zoonotic *B. pahangi* infection [8, 87, 88]. Soon after reporting a *B. pahangi* contracted case, a routine entomological survey relies on the establishment of a sentinel site suited to sampling local vector populations; this also includes a patient’s house. If the zoonotic *B. pahangi* infection is locally acquired through mosquito-borne transmission in certain place and time, two assumptions of the entomologic investigation of plantation-related *B. pahangi* filariasis in humans need to be logically analyzed. If the infection occurs in an area where *B. malayi* is endemic, *Ar. subalbatus* versus its counterparts such as *Mansonia uniformis*, *Mansonia indiana*, and *Cx. quinquefasciatus* are suspected to be vector-host preference of *B. pahangi*. If the infection occurs in an area where *B. malayi* is not endemic, *Ar. subalbatus* versus its counterparts, such as *Ae. togoi* and *Cx. quinquefasciatus* are suspected to be vectors of *B. pahangi*.

Such emerging *B. pahangi* infections occurring in non-transmission area of *B. malayi* in Thailand have been epidemiologically linked to be transmitted by locally adapted plantation ecotype of *Ar. subalbatus* vector. Based on appropriate collection methods and species identification, indoor and outdoor collections of female adult mosquitoes are needed. The availability of larval breeding sites for *Ar. subalbatus* is considered proximal to a patient’s house or distant within a 10–100-m radius of a patient’s house in an infection pocket. For instance, *Ar. subalbatus* elicits the infestation, that is, the abundance and distribution during its peak hour at night (Figure-6) for 2–3 consecutive days. Its infestation relates to adult vector abundance to the availability of larval breeding sites that elicit larval abundance surrounding a patient’s house.

Several plausible factors that influence the levels of infestation or reinfestation of *Ar. subalbatus* include human settlements, household sanitation, the domestication of livestock or pet animals, and local environments suited to favor larval breeding. It is possible that if accompanied by sustaining the levels of *B. pahangi* infection prevalence in domestic cats and/or dogs, the degrees of *B. pahangi* infection prevalence in *Ar. subalbatus* are strongly associated with a unique ecotope of plantation-related *B. pahangi* filariasis [8]. Nonetheless, any *B. pahangi* contracted patient may or may not relate the infection to keeping close contact with pet animals such as cats or dogs in impoverished patient’s house.

## Conclusion

After the termination of the elimination (or interruption of transmission) of *B. malayi* or *W. bancrofti* in humans in endemic countries implementing the national program to eliminate lymphatic filariasis, the program coordinators and allied sectors need to understand the likelihood that local inhabitants residing in either transmission or non-transmission areas are at risk of, or affected with, the zoonotic *B. pahangi* infections if they are likely to have living or working conditions with the socio-ecological and biological vulnerability. Any local inhabitants who may carry the zoonotic *B. pahangi* infection in their lifetime are more likely to be infants and preschool-age children than school-age children and adults. As for case surveillance or investigation, any *B. pahangi* patient-cases who are diagnosed using serological and parasitological diagnostic methods are laboratory-confirmed by molecular marker-based PCR assays. However, the PCR-positive results do not differentiate between the clonal and multiple clonal PCR products that infer the spillover of zoonotic *B. pahangi* parasite populations circulating in any foci.

## Authors’ Contributions

AB: Conceived the concepts of plantation-related zoonotic *B. pahangi* filariasis and original artworks or graphic illustration. AB, PN, AI, SP, and WR: Provided current knowledge of *B. pahangi* life cycle, vector’s life cycle and current state of research on the epidemiology and ecology of *B. pahangi* zoonosis. AB and AI: Conceived the diagnostic approaches to detecting and differentiating *B. pahangi* and leveraged the bioinformatics. AB, PN, SP, and WR: Conceived and leveraged the data/information of *Ar. subalbatus*. All authors have read, reviewed, and approved the final manuscript.
